# Potential Candidates for Emergency Department Initiated Extracorporeal Cardiopulmonary Resuscitation (ECPR) in a Canadian Institution

**DOI:** 10.7759/cureus.29318

**Published:** 2022-09-19

**Authors:** James Gould, Judah Goldstein, Andrew H Travers, Janel M Swain, Alix Carter, Derek Rollo, Jay Mekwan, Paul Atkinson, George Kovacs

**Affiliations:** 1 Emergency Medicine, Queen Elizabeth II Health Science Center/Dalhousie University, Halifax, CAN; 2 Emergency Medicine, Dalhousie University, Halifax, CAN; 3 Emergency Medicine, Emergency Health Services, Dartmouth, CAN; 4 Family Medicine, Saint John Regional Hospital, Saint John, CAN; 5 Emergency Medicine, Horizon Health Network, Saint John, CAN; 6 Emergency Medicine, Saint John Regional Hospital, Saint John, CAN

**Keywords:** out-of-hospital cardiac arrest, emergency medical service, cardiac arrest, extracorporeal cardiopulmonary resuscitation, extracorporeal membrane oxygenation

## Abstract

Introduction

Out-of-hospital cardiac arrest (OHCA) patients experience poor survival. The use of extracorporeal membrane oxygenation (ECMO), a form of heart-lung bypass, in the setting of cardiac arrest, termed extracorporeal cardiopulmonary resuscitation (ECPR), has promise in improving survival with good neurologic outcomes. The study objective was to determine the number of potential annual ECPR candidates among the OHCA population in a health region within the Atlantic Canadian province of Nova Scotia.

Methods

A retrospective chart review was conducted over a five-year period: January 1st, 2012 to December 31st, 2016. Consecutive non-traumatic OHCA and emergency department (ED) cardiac arrests occurring in a pre-determined catchment area (20-minute transport to ECMO center) defined by a geographic bounding box were identified. Criteria for ECPR were developed to identify candidates for activation of a “Code ECPR”: (1) age 16-70, (2) witnessed arrest, (3) no flow duration (time to CPR, including bystander) <10 minutes, (4) resuscitation >10 minutes without return of spontaneous circulation (ROSC), (5) emergency medical service (EMS) transport to hospital <20 minutes, (6) no patient factors precluding ongoing resuscitation (do not resuscitate status (DNR), palliative care involvement, or metastatic cancer), and (7) initial rhythm not asystole. Candidates were stratified by initial rhythm. Candidates were considered ultimately ED ECPR eligible if they failed conventional treatment, defined by death or resuscitation >30 minutes. Clinical data related to candidacy was extracted by an electronic query from prehospital and ED electronic records and manual chart review by three researchers.

Results

Our search yielded 561 cases of EMS-treated OHCA or in-ED arrests. Of those 204/561 (36%; 95% CI 33-40%) met the criteria for activation of a “Code ECPR”. Ultimately 79/204 (34%; 95% CI 28-41%) of those who met activation criteria were considered ED ECPR eligible; which is 14% (95% CI 11-17%) of the total number of arrests-of the total number of arrests, the initial rhythms were pulseless electrical activity (PEA) 33/79 (42%; 95% CI 32-53%) and shockable 46/79 (58%; 95% CI 47-69%).

Conclusion

Of all cardiac arrests in the area surrounding our ECMO center, approximately 41 per year met the criteria for a Code ECPR activation, with 16 per year ultimately being eligible for ED ECPR. This annual estimate varies based on the inclusion of initial rhythm. This provides insight into both prehospital and hospital implications of an ED ECPR program and will help guide the establishment of a program within our Nova Scotian health region. This study also provides a framework for similar investigation at other institutions contemplating ED ECPR program implementation.

## Introduction

There are 40,000 cardiac arrests in Canada each year and approximately 85% of those arrests occur outside of hospitals [1]. Unfortunately, the current survival rate for out-of-hospital cardiac arrest (OHCA) in Canada is very low, estimated to be less than 10% [2,3]. The survival rate with good neurologic outcome is much lower for cases requiring prolonged resuscitation > 30 minutes, termed refractory cardiac arrest [4]. Fortunately, the use of extracorporeal membrane oxygenation (ECMO) in cardiac arrest, termed extracorporeal cardiopulmonary resuscitation (ECPR), has shown promise in observational studies, with neurologic outcomes as high as 30-40% in refractory cardiac arrest [5-20].

An increasing number of institutions have adopted processes and criteria for ECPR programs. While there is a paucity of data to determine the optimal candidate for an ECPR program, a number of general criteria are outlined which include refractory arrests of young patients with witnessed arrests, and no flow or time to CPR is expected to be short [4,21]. A study into the optimal timing of ECPR initiation has been conducted and shows that as CPR duration (low flow time) exceeds one hour, the chance of good neurologic outcome with ECPR diminishes [22]. A recently published strategy for program implementation in a tertiary care center in Vancouver, British Columbia showed that the establishment of an ECPR program for OHCA, that used prehospital activation of a multidisciplinary emergency department (ED) ECPR team, termed a “Code ECPR”, reduced the interval to ECMO flows by half; 120 minutes to 60 minutes [23]. Timing of patient transport from the prehospital setting in order to meet this 60-minute threshold is important. The optimal transport interval without diminishing the chance of prehospital return of spontaneous circulation (ROSC), is approximately 8-24 minutes [24-25].

Given the narrow window of time available for delivery of ECPR for OHCA, a well-organized ED ECPR program is required. The allocation of resources to such a program must be justified by the benefit. In order to determine the benefit, the primary question must be the need. As such the first question that should be answered is how many potential candidates there may be, on a yearly basis in the region in question. The aim of this study is to determine the number of potential yearly ECPR candidates within a Nova Scotian health region, to help inform the establishment of an ECPR program.

## Materials and methods

Design and setting

A five-year retrospective chart review (January 1st, 2012 to December 31st, 2016) was conducted in our health region. This health region encompasses the Halifax Regional Municipality (HRM) in the metropolitan area of Halifax, Nova Scotia. The area has a metropolitan population of roughly 400,000 people. The study region, a smaller portion of the HRM, includes the only tertiary care facility and ECMO center in the province (Halifax Infirmary) as well as two smaller hospitals each with their own ED.

Emergency medical services (EMS) in Nova Scotia, including ground and air ambulance, medical communications center, and first response, are delivered under a single contractor model termed Emergency Health Services (EHS). A staffing mix of primary, intermediate, and advanced care paramedics work in the ground ambulance system in a single agency. The EMS system in this region has evidence-based clinical practice guidelines (CPG) that are updated regularly based on appraised literature. The evidence-based CPG for cardiac arrest includes high-quality CPR, typical advanced life support (ALS) interventions, and post ROSC care [26]. The CPG also includes guidelines for on-scene termination of resuscitation and transport. Cardiac arrests in the study region all have ALS response with some receiving medical first responder or basic life support (BLS) prior to ALS arrival. The study was approved by the Nova Scotia Health research ethics board (REB file #: 1022517).

Selection of participants

All non-traumatic OHCA or in-ED arrests within our study setting during the study period were identified. Based on previously suggested ECPR criteria from established ECPR programs and local practice, criteria were developed to identify potential candidates for prehospital or ED activation of a “Code ECPR” which would alert a specialized team to prepare for ECPR delivery in the ED: (1) age 16-70, (2) witnessed arrest, (3) no flow duration (time to CPR, including bystander) <10 minutes, (4) resuscitation >10 minutes without ROSC, (5) EMS transport to hospital <20 minutes, (6) no patient factors precluding ongoing resuscitation (do not resuscitate status (DNR), palliative care involvement, or metastatic cancer), and (7) initial rhythm not asystole [3,4]. Those patients who met “Code ECPR” activation criteria were then determined whether they were eligible for ED ECPR. This meant that they may have hypothetically been placed on ECMO once they arrived in the ED if a program had existed. Patients were ED ECPR eligible if they failed conventional treatment, defined by death or resuscitation >30 minutes.

Data

All EHS patient documentation is done by tablet into an electronic patient care record (ePCR), which is stored in an electronically queryable database. A pre-existing cardiac arrest case finding algorithm was used to find all non-traumatic cardiac arrests in this provincial database. This was then limited to OHCA that occurred within our study region using call coordinates (longitude and latitude) within a geographic bounding box. This bounding box included locations that were an estimated 20-minute transport time to the ECMO center. Statistics Canada defines the “Population Centre” of Halifax within the bounding box. According to 2016 census data, the population density was 1349.3 per square kilometer [27]. Our population density would be lower considering the bounding box is slightly larger than the Population Center, though this provides a rough comparison to other systems. All ED cardiac arrests occurring within any of the three study setting EDs were identified through the electronic ED database. Patient characteristics and arrest variables were electronically extracted from each case. Any missing data was extracted by excepted methods of manual review by three researchers, the principal investigator and two research assistants. The principal investigator conducted a quality check of any chart flagged for ambiguous or incomplete information reviewed by the research assistants.

Data analysis

The data was analyzed using Microsoft Excel 2008 and GraphPad for confidence intervals by the modified Wald method. The collected variables were reported as percentages and 95% confidence intervals for dichotomous variables and either means with standard deviations or medians with interquartile ranges (IQRs) for continuous variables.

Outcome

The primary outcome of this study was the number of patients with OHCA or ED cardiac arrest in this particular health region that may have been potential candidates for an ED ECPR program. All potential candidates were further stratified based on initial cardiac rhythm on health professional monitor; (1) pulseless electrical activity (PEA) and (2) shockable (ventricular fibrillation (VF)/ventricular tachycardia (VT)). Given the variability of included rhythms in current ECPR programs, this stratification allowed for improved decision-making with regard to rhythm inclusion during ECPR program establishment.

## Results

Patient and arrest characteristics

Within the study period and geographic 20-minute transport bounding box, there were 561 EMS-treated OHCA or in-ED arrests (Table 1). The median age was 58 (IQR 51-65), with a predominance of male patients 396 (70%).

**Table 1:** Patient characteristics based on demographic and past medical history IQR: interquartile range; PMHx: past medical history

		n (IQR or %)
Demographics	Total cases	561
	Age (median)	58 (51-65)
	Male	396 (70)
	Female	164 (30)
	Unknown (Male/Female)	1 (0.17)
PMHx	Myocardial Infarction	114 (20)
	Coronary Artery Bypass Graft	41 (7.3)
	Pace Maker	16 (2.9)
	Hypertension	251 (45)
	Diabetes Mellitus	161 (29)
	Congestive Heart Failure	69 (12)
	Atrial Fibrillation	36 (6.4)
	Chronic Obstructive Pulmonary Disease	97 (17)
	Chronic Liver Failure	15 (2.7)
	Chronic Renal Failure	40 (7.1)
	Stroke	30 (5.3)
	Metastatic Cancer	36 (6.4)
	Do Not Resuscitate Order	36 (6.4)
	Palliative Care Patient	38 (6.8)

Main results

Arrest characteristics for all OHCA and in-ED cardiac arrest cases are shown in Table 2. There was a high rate of witnessed arrests 396/561 (71%) and bystander CPR 406/561 (72%). This included arrests in the ED, which were assumed to be witnessed with bystander CPR. The most common initial rhythm was asystole 234/561 (42%) followed by shockable rhythms 204/561 (36%) and PEA 119/561 (21%). When the cardiac arrest was witnessed, no flow time was <10 minutes in 382/561 (68%). Additional interventions were provided in the majority of cases; any epinephrine 478/561 (85%) with mean number of epinephrine doses per patient of 4 (IQR 3-5); any defibrillation in 277/561 (49%) with mean number of defibrillations per patient of 2 (IQR 1-5); amiodarone was given less frequently 86/561 (15%).

**Table 2:** Cardiac arrest characteristics for all out-of-hospital and in-emergency department cardiac arrest cases IQR: interquartile range; CPR: cardiopulmonary resuscitation

		n (IQR or %)
Witnessed		396 (71)
Bystander CPR		406 (72)
Initial Rhythm	Asystole	234 (42)
	Pulseless Electrical Activity	119 (21)
	Shockable	204 (36)
	Unknown	4 (0.71)
No Flow Time (min)	N/A (unwitnessed)	152 (27)
	0-2	334 (60)
	2-4	9 (1.6)
	4-6	15 (2.7)
	6-8	7 (1.2)
	8-10	17 (3.0)
	10-15	9 (1.6)
	15-20	8 (1.4)
	>20	10 (1.8)
Any Epinephrine		478 (85)
Number of Epinephrine Doses		4 (3-5)
Any Defibrillations		277 (49)
Number of Defibrillations		2 (1-5)
Amiodarone Given		86 (15)
	1 dose	66 (12)
	2 doses	20 (3.6)

**Figure 1:**
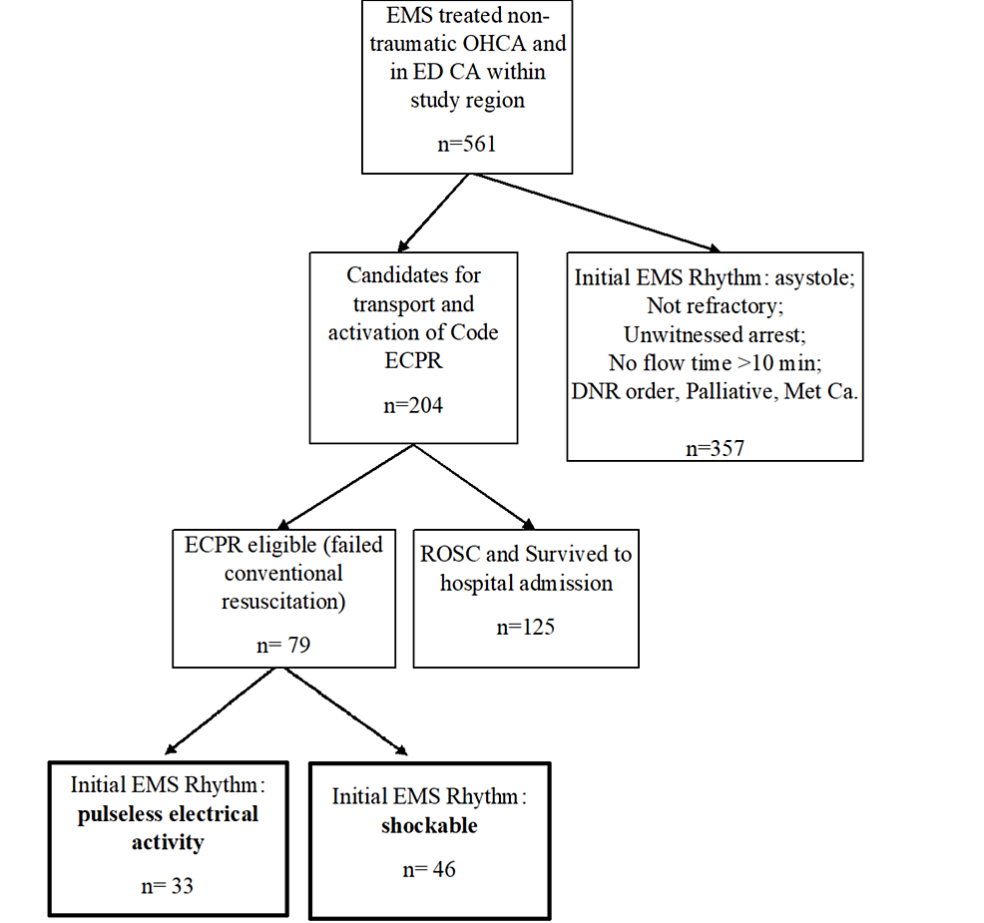
Flow of patients showing candidates for ED ECPR program with identification of initial EMS rhythm EMS: emergency medical service; OHCA: out-of-hospital cardiac arrest; ED: emergency department; CA: cardiac arrest; ECPR: extracorporeal cardiopulmonary resuscitation; DNR: do not resuscitate; Met Ca: metastatic cancer; ROSC: return of spontaneous circulation

Of the 561 cases, 204/561 (36%; 95% CI 33-40%) met the criteria for activation of a “Code ECPR” (Figure 1). Ultimately, 79/204 (34%; 95% CI 28-41%) of those who met activation criteria were considered ED ECPR eligible; which is 14% (95%; CI 11-17%) of the total number of arrests. Of all ED ECPR-eligible patients, PEA represented 33/79 (42%; 95% CI 32-53%) and shockable 46/79 (58%; 95% CI 47-69%) as the initial rhythms.

## Discussion

Interpretation

Based on our five-year retrospective chart review, our institution would expect up to 41 “Code ECPR” activations each year, or approximately 3.4 per month. Of those patients, approximately 16 per year or 1.3 per month would ultimately be placed on ECMO based on the failure of conventional resuscitative efforts. This would yield an approximate rate of activation to procedural completion of 39%.

Previous studies

A recent study conducted on an Australian ECPR program showed a good neurologic outcome rate of 33%. The cost per patient incurred, which included the sum of all money attributed to the patient’s prehospital and hospital stay (including but not limited to consumables, medications, staffing, and ICU and ward costs), was $56 889 CAD. Based on their neurologic outcome rate, this equated to $23 750 CAD per quality-adjusted life year (QALY) gained. The American College of Cardiology (ACC) and American Heart Association (AHA) statement on cost/value consider any cost/QALY gained <$150 000 to be cost-effective, with any cost/QALY <$50 000 as being of high value. As such, despite seemingly high cost, ECPR for OHCA can be considered very cost-effective. Furthermore, many of the items included in this particular analysis were fixed costs (staffing, ICU/ward costs). The cost per patient is reduced to $20 841 CAD when only ECMO-related costs are considered [28]. Considering the results of our needs assessment these costs would equate to approximately $329 382 CAD per year locally.

Limitations

A number of assumptions were made that may have both underpredicted and overpredicted candidate numbers. Firstly, we assumed that any patient achieving ROSC and surviving to hospital admission were excluded from requiring ECPR, as a surrogate of refractory cardiac arrest. This is a limitation of the retrospective design. In reality, a number of the patients who survived to hospital admission would have been ECPR candidates based on the duration of their cardiac arrest. However, we may have simultaneously overpredicted the potential candidate number by the minimal set of exclusion criteria used in this study, again limited by the retrospective design. In reality, a number of additional exclusion criteria would have been applied to this patient population that was not available for analysis in all patients in this study population, most notably; pH and lactate values, body mass index, and severity of baseline debilitating medical conditions.

Finally, we used a geographic bounding box as a surrogate of transport interval that would allow for ECMO flows within an adequate time frame. The existing evidence suggests that the most benefit from ECPR comes with initiation at <60 minutes of conventional CPR. Given this was a retrospective review, we have no data on the time it would take to cannulate the patients included in our study, and due to current protocols of on-scene termination for refractory cardiac arrests, we have an absence of data on transport intervals for a large number of patients. As such, the only reliable surrogate was the geographic location of the arrest, which predicted potential transport time. However, geographic location does not account for difficult extrications that may account for prolonged transport intervals despite close proximity.

Research implications

A well-established literature base does not currently exist to guide the optimal number of patients required for an ED-based ECPR program. A position paper has made suggestions for the minimal number of extracorporeal life support (ECLS) cases that should be performed in an institution to maintain proficiency which ranges from 20-30 cases [29]. While our predicted number of ED ECPR is below this at 16, this does not take into account the number of ECLS cases performed at the institutional level. In our institution, as with many others initiating an ED ECPR program, the multidisciplinary team involved includes individuals that are involved and participate in ECMO cases in the ICU for cardiac failure, respiratory failure, and in-hospital cardiac arrest. Therefore, while the total number of ED ECPR cases does not meet the recommended number of ECLS cases to maintain a program, the institutional program likely does. Furthermore, this does not take into account the number of ECLS simulation scenarios performed for each institution. An institution that has developed a highly functional ECMO simulation program may operate at a highly functional level with their ED ECPR program despite limited case numbers. Finally, there is certainly valuable practice, development, and maintenance of proficiency associated with ED ECPR team activations that do not proceed to ECMO completion. A great deal of skill acquisition and proficiency will come from multidisciplinary team organization, which should occur for all 41 annual activations, regardless of whether the case proceeds to completion.

There is no literature currently to guide the optimal percentage of ECPR activations that proceed to ECPR initiation. The provision of ECPR in the ED is a high acuity low opportunity scenario that requires streamlined function of a multidisciplinary team. The goal of the program should be to activate often enough that the multidisciplinary team involved has enough exposure to the process, but not so often to overwhelm the resources required for activations.

Future studies should examine the optimal number of ED ECPR patients required per year to maintain a proficient program, the optimal percentage of team activations that proceed to completion of ECMO, how to obtain that percentage, and finally how simulation-based training contributes to those recommendations.

Finally, many of the EMS treated OHCA in this study who were deemed candidates for ED ECPR activation and transport, in reality had their resuscitations terminated on scene and were thus not transported under a model of on-scene resuscitation. With the introduction of an ED ECPR program, the corresponding EMS system should expect an increase in their number of OHCA transports. This has significant implications for the prehospital system of care. In anticipation of this, EMS systems should ensure that there is adequate training and resources for paramedics to optimize the process of patient identification, extrication, and transport, and to ensure the safety of the provider during that process. Therefore, the involvement of EMS stakeholders and senior leadership at every level of ED ECPR program planning is of upmost importance.

Based on our predicted patient numbers, and on existing literature that suggests rates of good neurologic outcomes of up to 30-40% with ECPR, our institution could expect up to five to six additional cardiac arrest survivors with good neurologic outcomes. Given that these are generally young patients, this small number still has the potential to provide a meaningful difference for both the individual patient and society.

## Conclusions

Of all cardiac arrests in the area surrounding our ECMO center, approximately 41 per year met the criteria for a Code ECPR activation, with 16 per year ultimately being eligible for ED ECPR. This annual estimate varies based on the inclusion of initial rhythm. This will help guide the establishment of an ED ECPR program in our Nova Scotian health region, which based on the existing outcome data for ECPR has the potential to provide a small but meaningful impact on cardiac arrest outcomes in this region. Furthermore, this provides a comparator group for future research locally post-program implementation, as well as an investigational framework for other institutions preparing to implement their own ED ECPR programs.
